# Sleep Quality, Circadian Rhythm Stability and Changes in Delirium State in Predicting Mortality Risk in Intensive Care Unit Patients: A Prospective Observational Study

**DOI:** 10.1111/nicc.70241

**Published:** 2025-11-17

**Authors:** Hui‐Ju Lai, Wen‐Pei Chang

**Affiliations:** ^1^ Department of Nursing, Shuang Ho Hospital Taipei Medical University New Taipei City Taiwan; ^2^ School of Nursing, College of Nursing Taipei Medical University Taipei Taiwan

**Keywords:** circadian rhythm, delirium, intensive care unit, mortality, sleep quality

## Abstract

**Background:**

Delirium is a common neuropsychiatric complication in the intensive care unit (ICU), the occurrence of which is closely associated with patient prognoses.

**Aim:**

To examine the associations between sleep quality and circadian rhythm stability measured by actigraphy and changes in delirium state in intensive care unit (ICU) patients, as well as their predictive power for mortality risk.

**Study Design:**

ICUs of a medical centre in Taiwan, from September 1, 2024, to January 31, 2025. A prospective observational study was conducted in adult ICU patients. Wrist‐worn actigraphy was used to monitor total sleep time (TST, h), wake after sleep onset (WASO, h), and circadian rhythm stability (24‐h autocorrelation coefficient, *r*24) for 72 consecutive hours. Delirium was assessed twice daily for three days using the Confusion Assessment Method for the ICU (CAM‐ICU) and categorized as no delirium, prolonged delirium (lasting ≥ 3 days), or new‐onset delirium (developed after enrollment).

**Results:**

A total of 74 ICU patients were included. Among them, 30 had no delirium, 20 had prolonged delirium, and 24 developed new‐onset delirium. Mortality rates in both the prolonged delirium and new‐onset delirium groups were 45%, significantly higher than in the no‐delirium group (13.3%, *p* = 0.015). The prolonged delirium group had higher Sequential Organ Failure Assessment (SOFA) scores, longer WASO and lower *r*24 than the no‐delirium group, with *r*24 significantly associated with prolonged delirium (OR = 0.001, *p* = 0.012). The new‐onset delirium group showed higher WASO, which was significantly associated with delirium (OR = 1.04, *p* = 0.046). Multivariate Cox analysis identified prolonged delirium (HR = 3.92, *p* = 0.049) and SOFA score (HR = 1.32, *p* = 0.027) as independent predictors of mortality.

**Conclusions:**

WASO and *r*24 were closely linked to delirium state changes. Lower *r*24 was strongly associated with prolonged delirium and higher mortality, while higher WASO was related to new‐onset delirium.

**Relevance to Clinical Practice:**

Continuous monitoring of sleep continuity and circadian rhythms in ICU patients is recommended. Incorporating WASO into early delirium risk assessments may facilitate timely interventions, reduce delirium incidence and mortality and improve critical care quality.

**Impact Statements**

- What is known about the topic
  ○ Delirium is a common and serious complication among intensive care unit (ICU) patients and is strongly associated with worse outcomes, including higher mortality.
  ○ Poor sleep quality and disrupted circadian rhythms may contribute to delirium development, but few have objectively measured these factors using actigraphy in critically ill patients.
- What this paper adds
  ○ This study provides prospective evidence demonstrating that both sleep fragmentation and circadian rhythm instability, as measured by actigraphy, are associated with different delirium states among ICU patients.
  ○ Circadian rhythm instability was strongly linked to prolonged delirium and increased mortality risk, highlighting the prognostic value of actigraphy‐based monitoring in critical care.

## 1 Introduction

Delirium is an acute brain dysfunction characterised by decreased attention, disorientation and declines in memory, language and thinking abilities as well as by increased hallucinations or delusional psychiatric symptoms [1]. As a common and severe neuropsychiatric complication among intensive care unit (ICU) patients, the prevalence of delirium can reach 25%–74% among ICU patients [2, 3] and even as high as 60%–80% among mechanically ventilated ICU patients [3, 4]. Delirium occurrence reflects the vulnerability of the brain to systemic stress (e.g., infection, hypoxia, inflammation and metabolic imbalance) and is closely associated with the patient's overall physiological resilience [5]. Not only does the appearance of delirium prolong hospital stays and increase medical costs, but delirium is also closely associated with long‐term cognitive impairment and increased mortality rates [6].

The pathological mechanisms of delirium are extremely complex. A growing number of studies suggest that sleep disruption and circadian rhythm dysregulation play key roles in the development of delirium [7, 8]. Poor sleep quality, especially increases in wake after sleep onset (WASO), is a commonly used indicator of the degree of sleep fragmentation in ICU patients [9]. A recent study using actigraphy reported that increases in WASO are significantly correlated with acute brain dysfunction, including the occurrence of delirium [10]. Moreover, circadian rhythm stability indicates whether a patient's activity fits their physiological rhythms, and decreases in circadian rhythm stability are associated with abnormal melatonin secretion and neuroinflammation, which could render patients more susceptible to delirium [11]. However, limited research currently exists on the correlations between changes in delirium state with quantitative indicators of sleep disruption and circadian rhythm stability. Although the 24‐h autocorrelation coefficient (*r*24) is currently a crucial index of circadian rhythm stability in sleep and mental health research [12, 13], there remains a lack of empirical evidence on the use of *r*24 in ICU patients as a tool to explore correlations with delirium or disease prognosis.

**FIGURE 1:**
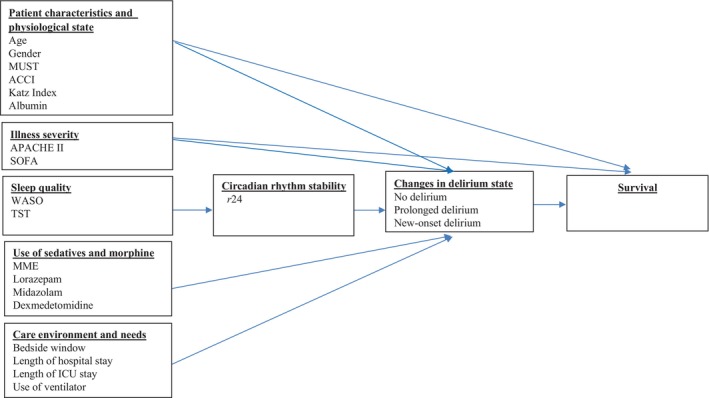
Research framework.

Most existing studies have divided patients into those with and without delirium [14], with no further investigation of clinical differences among delirium subtypes. Recent evidence suggests that the onset and trajectory of delirium have distinct prognostic implications; for example, prolonged delirium is strongly correlated with mortality risk [15]. Moreover, the severity of illness and the baseline physiological states of patients are recognised determinants of ICU outcomes, independent of intermediate factors such as delirium, sleep quality or circadian rhythm disruption. These variables, incorporated into prognostic scoring systems (e.g., APACHE II or SOFA), may influence survival directly and/or indirectly through neurobehavioral pathways [16, 17, 18, 19]. Building on this evidence, the present study examined the correlations among sleep quality, circadian rhythm stability and delirium trajectories in ICU patients as well as the association of these variables with mortality. We developed an integrated research framework (Figure 1) that links physiological status, environment, sedative use and neurobehavioral manifestations to patient outcomes, thus providing a basis for the assessment of evidence‐based ICU prognosis. Accordingly, this study aimed to determine how actigraphy‐measured sleep and circadian rhythm indicators are associated with different delirium trajectories and mortality among critically ill patients

## 2 Design and Methods

### 2.1 Design and Participants

We adopted a prospective observational study design with adult ICU patients at a medical centre in northern Taiwan as participants. The centre included four adult ICUs (medical, surgical, neurological/neurosurgical and cardiology/cardiovascular surgery) with a total of 80 beds. Patients admitted between September 1, 2024, and January 31, 2025, were screened for eligibility. The inclusion criteria were age over 20 years, a Richmond Agitation‐Sedation Scale (RASS) score over −4 (meaning that the patient could be aroused by voice), and the ability to communicate in Mandarin Chinese or Taiwanese Hokkien. Their attending physician also had to anticipate that the patient would require admission and care in the ICU for at least 72 h. Patients who were expected by their attending physician to pass away within 72 h, whose delirium symptoms could not be evaluated (e.g., severe hearing or visual impairment), who had already been diagnosed with insomnia or psychiatric disorders such as dementia or who were uncooperative prior to being admitted to the ICU, or who had circadian rhythm dysregulation or delirium induced by brain damage (e.g., brain tumours or brain surgery) were excluded. All ICU rooms were single occupancy with uniform configuration and environmental control. Approximately 30% of the rooms had external windows providing natural light, while the remaining rooms did not have a window and thus did not have exposure to natural light. Environmental factors such as lighting (1000–2000 lx from 06:00 to 21:00), temperature and noise levels were standardised across all ICUs. The presence or absence of a window thus served as a proxy indicator of light exposure.

### 2.2 Sample Size

We employed G*Power 3.1 to estimate the number of samples needed and used the Log‐rank test to simulate the number of samples needed for a Cox proportional hazards model. The estimation conditions included a type I error (α) set to 0.05 (two‐tailed) and a power of 0.80. According to previous research, the mortality risk of individuals with delirium is 2.2 times higher than that of individuals with no delirium; thus, the hazard ratio (HR) is 2.2 [20]. With a 1:1 allocation ratio, we estimated that the mortality rate of critically ill patients during hospitalisation would be 25%. Based on the above parameter settings, we calculated that 50 samples in total would be required.

### 2.3 Data Collection

Data were collected at three major time points: baseline (enrolment), ICU observation (Days 1–3) and six‐month follow‐up, as summarised in Table 1.

**TABLE 1:** Summary of data collection time points and variables.

Time point	Variables collected	Source/method
Enrolment (baseline)	Demographics (age, gender, BMI, diagnosis, medical history, the number of days from hospital admission to study enrolment, the number of ICU days from ICU admission to enrolment); albumin (within 72 h prior to enrolment); ventilator use status; window position; severity indices (SOFA, APACHE II, ACCI, MUST, Katz)	Electronic medical records
ICU Days 1–3	Actigraphy parameters (TST, WASO, *r*24); sedative/analgesic dosages (MME/day); CAM‐ICU scores and delirium classification	Wrist‐worn device and chart review
6‐month follow‐up	Survival status (date of death or censoring)	Electronic medical records and outpatient records

Abbreviations: ACCI, age‐adjusted Charlson comorbidity index; APACHE II, acute physiology and chronic health evaluation II; CAM‐ICU, confusion assessment method for the ICU; MME, morphine milligram equivalents; MUST, malnutrition universal screening tool; *r*24, 24‐h autocorrelation coefficient; SOFA, sequential organ failure assessment; TST, total sleep time; WASO, wake after sleep onset.

At baseline, demographic, clinical and environmental characteristics were extracted from electronic medical records, including disease‐severity indices, laboratory values and other relevant variables such as age, body mass index, gender, primary diagnosis at ICU admission, medical history, the number of days from hospital admission to study enrolment, the number of ICU days from ICU admission to enrolment, albumin level and ventilator use status on the enrolment day.

During the ICU observation period, patients wore wrist‐worn actigraphs continuously for 72 h to record sleep–wake activity, and delirium was assessed twice daily using the Confusion Assessment Method for the ICU (CAM‐ICU). Sedatives and analgesics administered during this period, including lorazepam, midazolam, dexmedetomidine, morphine and fentanyl, were recorded daily. Lorazepam, midazolam and dexmedetomidine were documented by daily dosage (mg/day), whereas morphine and fentanyl dosages were converted to morphine milligram equivalents per day (MME/day) for standardised comparison and analysis [21].

Follow‐up data on six‐month overall survival were obtained from both inpatient and outpatient electronic medical records. If a patient died within the 6‐month follow‐up period, the date of death was defined as the endpoint; if the patient survived, the date on which a full 6 months was reached served as the censoring point. Patients were followed until July 31, 2025. For cases lost to follow‐up, if their last medical record indicated survival, the survival time was censored at the date of their last recorded clinical visit. The following subsections describe the assessment tools used to measure patient sedation, organ function, nutritional status, comorbidities and functional independence.

#### 2.3.1 Richmond Agitation‐Sedation Scale (RASS)

The RASS was used to assess sedation and arousal status, and only patients with RASS scores between −3 and +4 were further evaluated with the CAM‐ICU [22]. The RASS has demonstrated strong inter‐rater reliability across diverse ICU populations, including medical, surgical, cardiac surgical and neurological patients, with or without mechanical ventilation and sedative use (*r* = 0.922–0.983; kappa = 0.64–0.82) [23].

#### 2.3.2 Sequential Organ Failure Assessment (SOFA)

The SOFA score was used to evaluate the degree of organ dysfunction and prognosis in ICU patients. SOFA incorporates six major organ systems (respiratory, coagulation, hepatic, cardiovascular, neurological and renal), with higher scores indicating more severe organ dysfunction and increased risk of mortality. It has been widely applied in critical care research and clinical practice as a reliable prognostic tool [24, 25, 26].

#### 2.3.3 Acute Physiology and Chronic Health Evaluation II (APACHE II) Score

The APACHE II score was used to assess the severity of illness in ICU patients. This system integrates three components: an acute physiology score derived from vital signs and key laboratory variables, an age score and a chronic health score reflecting pre‐existing comorbidities. The total score provides an overall index of disease severity, with higher values associated with an increased risk of poor outcomes. The APACHE II has been extensively validated and remains one of the most widely used severity scoring systems in critical care research and practice [17, 27].

#### 2.3.4 Malnutrition Universal Screening Tool (MUST)

Developed by the British Association for Parenteral and Enteral Nutrition in 2003, the MUST was used to screen for the risk of malnutrition. It has been widely applied in hospitalised and elderly populations. Compared to the Patient‐Generated Subjective Global Assessment (PG‐SGA), MUST is better at detecting malnutrition, with a sensitivity of 69.7%, specificity of 75.8%, positive predictive value of 75.4% and negative predictive value of 70.1%; the kappa value of MUST is 72.7%, demonstrating acceptable reliability and validity [28, 29].

#### 2.3.5 Age‐Adjusted Charlson Comorbidity Index (ACCI)

The ACCI was used to evaluate the severity of comorbidities and their impact on patient prognosis. The ACCI is an extension of the original Charlson Comorbidity Index (CCI), which assigns weighted scores to a range of chronic diseases in order to quantify overall comorbidity burden. To improve prognostic accuracy, particularly in critically ill populations, the ACCI incorporates age as an additional weighted factor. This adjustment enables the index to reflect not only the presence of comorbidities but also the age‐related increase in mortality risk. The ACCI has been widely applied in studies of ICU patients and has consistently demonstrated good predictive performance for long‐term mortality [16, 30].

#### 2.3.6 Katz Index of Independence in Activities of Daily Living

The Katz Index of Independence in Activities of Daily Living was used to evaluate patients' functional status and level of independence. Originally developed by Katz in 1963, this tool has been widely applied in elderly and functionally impaired populations and demonstrates good internal consistency (Cronbach's α = 0.82) [31, 32].

#### 2.3.7 Confusion Assessment Method for the Intensive Care Unit (CAM‐ICU)

Developed by Ely et al., the CAM‐ICU was used to assess delirium in critically ill patients [33]. Patients with a RASS score of at least −3 were eligible for assessment. Delirium assessments were conducted twice daily, at 08:00 in the morning and 20:00 in the evening, using the CAM‐ICU for 3 consecutive days. The CAM‐ICU demonstrates good internal consistency in ICU populations, with Cronbach's α ranging from 0.82 to 0.86 [34, 35]. Based on CAM‐ICU results obtained over the three‐day assessment period, patients were classified into three groups. Prolonged delirium was defined as delirium present on all 3 days. New‐onset delirium was defined as the absence of delirium on day 1 and the morning of Day 2, with delirium emerging on the night of Day 2 or the morning of day 3 and persisting until the end of Day 3 assessments [36, 37]. Patients without delirium during all 3 days were classified as the no‐delirium group, and those not meeting these criteria were excluded.

#### 2.3.8 Actigraphy

The actigraph used in this study (Ambulatory Monitoring Inc., Ardsley, New York) was a wrist‐worn accelerometer in zero‐crossing mode (ZCM) that recorded movements per minute to infer sleep–wake states. Compared with polysomnography, its sensitivity, specificity and overall accuracy for sleep/wake classification are 84.9%, 74.2% and 79.0%, respectively. Given its ease of use and ability to continuously record, actigraphy has been increasingly applied in ICU patients [38, 39, 40].

Actigraphy served as the primary sleep evaluation tool in this study and was paired with sleep logs to obtain the total sleep time (TST), wake after sleep onset (WASO) and circadian rhythm stability index (*r*24) of the patients. Sleep research on ICU patients has shown that conventional actigraphy sleep parameters such as sleep efficiency (SE) can overestimate actual sleep. ICU patients are frequently in a state of low activity while remaining conscious, due perhaps to the fact that they are often prescribed sedatives that make them less active but not fully asleep or that they may be inactive due to wrist restraints. All of these may result in relatively high SE [11]. WASO is therefore the parameter more recommended for adoption when actigraphy is employed to gauge the sleep conditions of ICU patients [41]. WASO reflects the total amount of time in minutes that a patient is awake after falling asleep and can therefore reflect the sleep disruption and poor sleep continuity in ICU environments [42]. Gottlieb et al. noted that WASO over 100 min can be regarded as significant sleep fragmentation [43]. TST is often jointly interpreted with WASO to describe the sleep quality and sleep continuity of patients [11].

This tool was also used to measure the daily sleep–wake rhythm parameters of ICU patients, and the recorded data were analysed using Action‐4. *r*24, the 24‐h autocorrelation coefficient, is a parameter commonly used in sleep research to gauge circadian rhythm stability. Representing the degree of autocorrelation in an individual's activity patterns within a 24‐h period, *r*24 compares the strength of activity at a time point on two consecutive days. The level of consistency is used to assess whether changes in activity are repetitive and regular and follow a stable 24‐h cycle [44]. The value of *r*24 ranges from −1 to 1, with a higher value indicating that the patient's daily routine is more regular and that their circadian rhythm is more stable. *r*24 is a suitable indicator for monitoring the circadian rhythms of ICU patients because it can reflect circadian rhythm stability and facilitate assessments of the impact of the environment on the circadian rhythms of ICU patients [40]. In a study conducted by Jaiswal et al., *r*24 ≥ 0.35 served as a reference threshold for relatively stable circadian rhythms [45].

During the 72‐h actigraphy monitoring period, any use of restraints was documented in nursing records. Among the 74 patients, 53 (71.6%) experienced intermittent wrist restraint at some point, whereas 21 (28.4%) were not restrained. Note that the actigraph was consistently worn on the non‐restrained wrist. To examine whether restraint use affected actigraphy‐derived parameters, a Mann–Whitney *U* test was applied to compare the circadian rhythm stability index (*r*24) between the restrained and unrestrained groups.

#### 2.3.9 Sleep Logs

Actigraphy must be combined with sleep logs for the accurate analysis of sleep quality and circadian rhythm parameters. The sleep logs contained bedtimes and wake‐up times for three consecutive days, which the primary nurses recorded every day immediately after the patients fell asleep and woke up [46].

### 2.4 Ethical Considerations

This study was approved by the Taipei Medical University—Joint Institutional Review Board (TMU‐Joint IRB) (Review No.: N202404040) on April 30, 2024, and conducted in accordance with the Declaration of Helsinki and related ethical guidelines. This study was an observational study with no invasive measures. The details of this study were explained by the researchers or the primary nurses to the participants when their conditions were stable, and patients were only included in this study after written consent was obtained from the patients themselves or their legal representatives. In the consent forms, the researchers clearly expressed that participation was voluntary and that the participants could choose to withdraw at any time without affecting their healthcare rights. All collected data were anonymised to protect the privacy of the participants. The data were stored in a password‐protected computer, and only research team members could access and analyse the data as authorised. All physiological data, such as the sleep parameters, were managed in the same way to prevent data leaks. This study did not involve any financial incentives or risky interventions, and all procedures prioritised the respect and protection of patient rights.

### 2.5 Statistical Analyses

Data were analysed using IBM SPSS 25.0. Kolmogorov–Smirnov tests showed that most continuous variables (e.g., age, body mass index, MME, SOFA, APACHE II, ACCI, MUST, Katz Index, hospital/ICU stay, albumin, WASO and follow‐up period) were non‐normally distributed and thus presented as medians with ranges; categorical variables (e.g., gender, primary diagnosis, medical history, ventilator use, bedside window, sedative use and mortality) were summarised as frequencies and percentages. Group comparisons were conducted using chi‐square or Fisher's exact tests for categorical variables and the Kruskal‐Wallis H test for continuous variables, followed by Dunn's post hoc test for pairwise comparisons among the three delirium groups (no delirium, prolonged delirium, new‐onset delirium).

We examined correlations between sleep indices (TST, WASO), circadian rhythm stability (*r*24) and delirium state using linear and logistic regression, with *R* ^2^ as the evaluative metric. Multivariate logistic regression was then applied to identify factors associated with prolonged or new‐onset delirium (reference: no delirium), including TST, WASO, *r*24, MME, bedside window, hospital/ICU stay, ventilator use and sedative use. For regression analyses, lorazepam, midazolam and dexmedetomidine were dichotomised into ‘use vs. no use’ due to limitations in sample size and wide variability in daily doses. Mortality‐related factors were analysed using Cox proportional hazards regression; variables significant in univariate analysis were further tested in multivariate models to identify independent predictors. A two‐tailed *p* < 0.05 was considered significant.

## 3 Results

### 3.1 Characteristics and Physiological States of Patients

**FIGURE 2:**
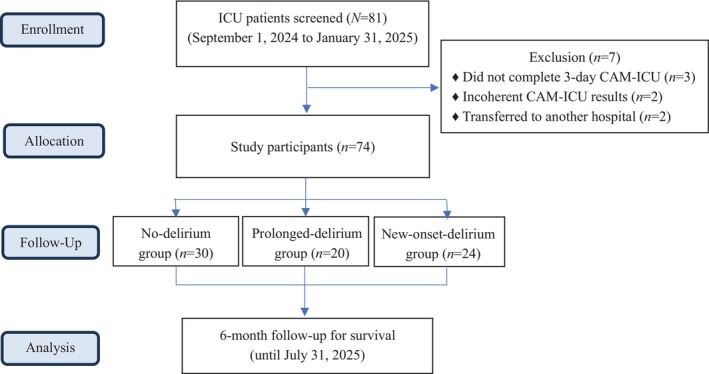
Flow diagram of patient screening, exclusion, group classification and six‐month survival follow‐up.

A total of 81 ICU patients were initially screened between September 1, 2024, and January 31, 2025. Following the exclusion of seven patients (three who did not complete the CAM‐ICU assessment for the full 3 days, two with incoherent assessment results and two who were transferred to another hospital and thus could not be followed up), 74 patients were ultimately included in the final analysis (Figure 2). The median age of the patients was 71 years (range 37–93), and the median body mass index was 24.8 kg/m^2^ (range 14.4–51.6); 62.2% were male. The main ICU admission diagnoses were acute respiratory failure (35.1%), septic shock (23.0%) and severe pneumonia (18.9%). Common comorbidities included hypertension (55.4%), diabetes mellitus (58.1%), coronary artery disease (31.1%) and chronic kidney disease (25.7%), with smaller proportions having cancer or other chronic illnesses. Most patients required ventilators (79.7%), and 29.7% had a bedside window. Sedative use was low, with 16.2% receiving lorazepam, 18.9% midazolam and 14.9% dexmedetomidine. The median opioid use was 25.0 mg MME/day (range 0–620). Illness severity was moderate to severe (SOFA median 6; APACHE II median 21). Nutritional and functional status were generally preserved (MUST median 0; ACCI median 4; Katz index median 6). The median hospital and ICU stays were 11.5 and 6 days, respectively, with albumin levels at 3.0 g/dL. Sleep and circadian assessments showed TST of 567.3 min, WASO of 93.5 min and *r*24 of 0.03. During follow‐up, 24 patients (32.4%) died, with a median follow‐up of 108 days (Table 2). A Mann–Whitney *U* test revealed no significant difference in r24 between the restrained and unrestrained groups (*p* = 0.149), indicating that restraint use did not significantly affect circadian rhythm stability.

**TABLE 2:** Comparison of demographic characteristics, clinical parameters and prognostic indices in three patient groups (*N* = 74).

Characteristic	Total	a. No delirium	b. Prolonged delirium	c. New‐onset delirium	*p*
	Median (range)	Median (range)	Median (range)	Median (range)	
*N* (%)	74	30 (40.5)	20 (27.0)	24 (32.5)	
Age (years)	71.0 (37–93)	69.5 (37–92)	66.5 (51–88)	73.5 (59–93)	0.415
Body mass index (kg/m^2^)	24.8 (14.4–51.6)	24.8 (14.8–51.6)	24.1 (16.9–43.3)	25.9 (14.4–44.1)	0.778
Male gender, *N* (%)	46 (62.2)	22 (73.3)	10 (50.0)	14 (58.3)	0.223
Primary diagnosis at ICU admission, *N* (%)					
Myocardial infarction	3 (4.1)	1 (3.3)	1 (5.0)	1 (4.2)	0.690
Cardiogenic shock	6 (8.1)	3 (10.0)	1 (5.0)	2 (8.3)	
Severe pneumonia	14 (18.9)	7 (23.3)	3 (15.0)	4 (16.7)	
Acute respiratory failure	26 (35.1)	6 (20.0)	10 (50.0)	10 (41.7)	
Acute respiratory distress syndrome	4 (5.4)	3 (10.0)	0	1 (4.2)	
Septic shock	17 (23.0)	7 (23.3)	4 (20.0)	6 (25.0)	
Upper gastrointestinal bleeding	2 (2.7)	2 (6.7)	0	0	
Diabetic ketoacidosis	2 (2.7)	1 (3.3)	1 (5.0)	0	
Medical history, *N* (%)					
Hypertension	41 (55.4)	17 (56.7)	11 (55.0)	13 (54.2)	0.982
Hyperlipidemia	10 (13.5)	3 (10.0)	2 (10.0)	5 (20.8)	0.443
Coronary artery disease	23 (31.1)	10 (33.3)	6 (30.0)	7 (29.2)	0.940
Atrial fibrillation or other arrhythmias	14 (18.9)	6 (20.0)	2 (10.0)	6 (25.0)	0.441
Diabetes mellitus	43 (58.1)	19 (63.3)	12 (60.0)	12 (50.0)	0.602
Chronic obstructive pulmonary disease	13 (17.6)	4 (13.3)	3 (15.0)	6 (25.0)	0.502
Stroke	6 (8.1)	1 (3.3)	1 (5.0)	4 (16.7)	0.171
Chronic kidney disease	19 (25.7)	5 (16.7)	5 (25.0)	9 (37.5)	0.219
End‐stage renal disease	9 (12.2)	3 (10.0)	3 (15.0)	3 (12.5)	0.867
Peptic ulcer disease	10 (13.5)	5 (16.7)	3 (15.0)	2 (8.3)	0.656
Liver cirrhosis	3 (4.1)	2 (6.7)	1 (5.0)	0	0.452
Solid tumour/haematologic malignancy	20 (27.0)	8 (26.7)	5 (25.0)	7 (29.2)	0.952
Ventilator, *N* (%)	59 (79.7)	22 (73.3)	18 (90.0)	19 (79.2)	0.355
Bedside window in ICU, *N* (%)	22 (29.7)	8 (26.7)	6 (30.0)	8 (33.3)	0.867
Sedatives and analgesics					
Use of lorazepam, *N* (%)	12 (16.2)	4 (13.3)	3 (15.0)	5 (20.8)	0.748
Use of midazolam, *N* (%)	14 (18.9)	6 (20.0)	5 (25.0)	3 (12.5)	0.563
Use of dexmedetomidine, *N* (%)	11 (14.9)	4 (13.3)	2 (10.0)	5 (20.8)	0.575
MME (mg MME/day)	25.0 (0–620)	41.0 (0–324)	0.67 (0–620)	29.0 (0–408)	0.769
SOFA score	6 (2–14)	5 (2–13)	8 (4–13)	6.5 (3–14)	0.002
APACHE II score	21 (5–40)	20 (5–32)	23 (10–40)	21.5 (12–39)	0.222
MUST score	0 (0–2)	0 (0–2)	0 (0–2)	0 (0–2)	0.213
ACCI	4 (0–10)	4 (0–10)	4.5 (1–8)	4.5 (2–8)	0.121
Katz index	6 (0–6)	4.5 (0–6)	6 (0–6)	3 (0–6)	0.165
Length of hospital stay (days)	11.5 (4–41)	12.5 (4–27)	14 (6–41)	9.5 (4–19)	0.074
Length of ICU stay (days)	6 (1–36)	5 (2–18)	7 (1–36)	5.5 (1–13)	0.349
Albumin (g/dL)	3.0 (2.0–4.0)	3.0 (2.1–3.9)	2.9 (2.0–3.6)	3.0 (2.4–4.0)	0.250
TST (min)	567.3 (420.7–703.5)	562.1 (420.7–691.5)	583.5 (423.7–703.5)	549.5 (435.0–643.0)	0.199
WASO (min)	93.5 (50.0–190.7)	87.6 (50.0–190.7)	108.3 (68.0–170.7)	105.2 (63.3–175.3)	< 0.001
*r*24	0.03 (−0.50–0.44)	0.17 (−0.19–0.44)	−0.07 (−0.50–0.20)	0.02 (−0.32–0.40)	< 0.001
Number of deaths, *N* (%)	24 (32.4)	4 (13.3)	9 (45.0)	11 (45.8)	0.015
Follow‐up period (days)	180 (20–180)	180 (51–180)	169 (20–180)	167 (26–180)	0.003

*Note:* Group comparisons of continuous variables were conducted using Kruskal–Wallis H tests; group comparisons of categorical variables were conducted using chi‐square tests.

Abbreviations: ACCI, age‐adjusted Charlson comorbidity index; APACHE II, acute physiology and chronic health evaluation II; MME, morphine milligram equivalents; MUST, malnutrition universal screening tool; *r*24, 24‐h autocorrelation coefficient; SOFA, sequential organ failure assessment; TST, total sleep time; WASO, wake after sleep onset.

### 3.2 Comparison of Characteristics and Physiological States of Patients in Three Delirium Groups

Based on delirium status, patients were divided into no delirium (*n* = 30), prolonged delirium (*n* = 20) and new‐onset delirium (*n* = 24) groups. The groups showed no significant differences in body mass index, primary diagnosis, medical history, demographics, ventilator use, bedside window, sedative use, opioid dosage, APACHE II, MUST, ACCI, Katz index, hospital/ICU stay, albumin or TST. However, mortality differed significantly (*p* = 0.015), being lowest in the no delirium group (13.3%) and highest in the prolonged (45.0%) and new‐onset (45.8%) groups (Table 2).

The three groups exhibited significant differences in SOFA score, WASO, *r*24 and follow‐up period (Table 3). Dunn's post hoc analysis revealed that the no delirium group had lower SOFA scores (*p* = 0.017, 0.004) and WASO (*p* = 0.001, 0.008) than the prolonged and new‐onset delirium groups, indicating more stable conditions and fewer sleep disruptions. For *r*24, only the no delirium and prolonged delirium groups differed significantly (*p* < 0.001), with greater circadian rhythm dysregulation in the latter. The follow‐up period was longer in the no delirium group than in the other two groups (*p* = 0.007, 0.025), whereas the prolonged and new‐onset groups showed no significant differences.

**TABLE 3:** Comparison of SOFA scores, WASO, *r*24 and follow‐up periods in three patient groups (*N* = 74).

Characters	Dunn's multiple comparisons test					
	a–b		a–c		b–c	
	H test value	*p*	H test value	*p*	H test value	*p*
SOFA score	−16.97	0.017	−18.75	0.004	−1.78	1.000
WASO (min)	−21.92	0.001	−17.71	0.008	4.21	1.000
*r*24	24.99	< 0.001	12.99	0.082	−12.00	0.196
Follow‐up period (days)	16.26	0.007	13.38	0.025	−2.88	1.000

*Note:* a. no delirium; b. prolonged delirium; c. new‐onset delirium.

Abbreviations: *r*24, 24‐h autocorrelation coefficient; SOFA, sequential organ failure assessment; WASO, wake after sleep onset.

### 3.3 Correlations Among Sleep Quality, Circadian Rhythm and Changes in Delirium State

Table 4 shows the correlations between sleep quality, *r*24 and delirium state. WASO was negatively correlated with *r*24 (*B* = −0.004, *p* < 0.001; *R* ^2^ = 34.7%), indicating that longer WASO reflected poorer circadian rhythm stability, whereas TST showed no significant correlation (*p* = 0.271). Logistic regression revealed that longer WASO increased the risk of both prolonged (OR = 1.04, *p* = 0.003) and new‐onset delirium (OR = 1.04, *p* = 0.008). *r*24 was significantly poorer in the prolonged delirium group (OR = 0.0004, *p* < 0.001) and was also associated with new‐onset delirium (OR = 0.02, *p* = 0.022), indicating that circadian rhythm dysregulation was linked to both delirium subtypes.

**TABLE 4:** Correlation among sleep quality, circadian rhythm and changes in delirium state (*N* = 74).

Independent variable	Dependent variable	*B*	95% CI	*p*	*R* ^*2*^
TST (min)^a^	*r*24	−0.0004	−0.001–0.0003	0.271	1.7%
WASO (min)^a^	*r*24	−0.004	−0.01 to −0.003	< 0.001	34.7%

Independent variable	Dependent variable	*B*	OR (95% CI)	*p*	χ^2^
TST (min)^a^	Prolonged delirium group^c^	0.01	1.01 (1.00–1.01)	0.306	3.98
	New‐onset delirium group^c^	−0.01	1.00 (0.99–1.00)	0.290	
WASO (min)^b^	Prolonged delirium group^c^	0.04	1.04 (1.01–1.07)	0.003	13.20
	New‐onset delirium group^c^	0.04	1.04 (1.01–1.06)	0.008	
*r*24^b^	Prolonged delirium group^c^	−7.84	0.0004 (0.00001–0.03)	< 0.001	19.53
	New‐onset delirium group^c^	−4.03	0.02 (0.001–0.56)	0.022	

Abbreviations: 95% CI, 95% confidence interval; *B*, unstandardised regression coefficients; OR, odds ratio; *R* ^2^, overall R square value; *r*24, 24‐h autocorrelation coefficient; TST, total sleep time; WASO, wake after sleep onset.

^a^ The explanatory power of the sleep variables with regard to *r*24 was explored using linear regression.

^b^ The predictive power of *r*24 with regard to changes in delirium state was analysed using logistic regression.

^c^ Reference category: no delirium group.

Table 5 examines factors associated with the delirium state. Compared with the no delirium group, only *r*24 was significant for prolonged delirium (OR = 0.001, *p* = 0.012), indicating that better circadian rhythm stability reduced the risk. Other factors, including WASO, TST, medication use, ventilator use, bedside window, opioid dosage and hospital/ICU stay, were not significant. For new‐onset delirium, WASO was significant (OR = 1.04, *p* = 0.046), suggesting that longer WASO increased the risk, while no other variables showed significant associations.

**TABLE 5:** Logistic regression analysis of factors correlated with prolonged delirium and new‐onset delirium (*N* = 74).

Predictors	Prolonged delirium		New‐onset delirium	
	OR (95% CI)	*p*	OR (95% CI)	*p*
TST (min)	1.003 (0.99–1.02)	0.566	1.00 (0.99–1.01)	0.611
WASO (min)	1.02 (0.98–1.06)	0.315	1.04 (1.001–1.08)	0.046
*r*24	0.001 (0.00001–0.22)	0.012	0.06 (0.001–7.37)	0.249
MME (mg MME/day)	1.00 (0.99–1.01)	0.802	1.00 (0.99–1.01)	0.885
Bedside window (Yes vs. No)	2.47 (0.47–13.03)	0.285	2.41 (0.56–10.44)	0.239
Length of hospital stay (days)	1.02 (0.86–1.22)	0.787	0.91 (0.76–1.09)	0.303
Length of ICU stay (days)	1.02 (0.84–1.23)	0.883	0.94 (0.75–1.16)	0.542
Use of ventilator (Yes vs. No)	0.96 (0.12–7.82)	0.967	0.87 (0.16–4.75)	0.868
Use of lorazepam (Yes vs. No)	0.88 (0.10–7.49)	0.903	1.12 (0.17–7.23)	0.907
Use of midazolam (Yes vs. No)	0.48 (0.07–3.51)	0.469	0.40 (0.06–2.82)	0.357
Use of dexmedetomidine (Yes vs. No)	1.29 (0.15–10.80)	0.817	3.12 (0.55–17.81)	0.200

*Note:* Reference category: no delirium group. Overall model summary: χ^2^ = 38.28, *p =* 0.017.

Abbreviations: 95% CI, 95% confidence interval; OR, odds ratio; *r*24, 24‐h autocorrelation coefficient; TST, total sleep time; WASO, wake after sleep onset.

### 3.4 Primary Factors Influencing Mortality Risk of ICU Patients

We then investigated the factors influencing the overall survival of the participants. Table 6 presents the results of Cox proportional hazards regression. In the univariate analysis, the SOFA score, APACHE II score, ACCI, WASO, *r*24 and changes in delirium state were all significantly correlated with mortality risk. Although the HR of ventilator use was 31.19 (*p* = 0.091), the correlation between ventilator use and mortality risk was not significant, due perhaps to the overly wide confidence interval (95% CI = 0.58–1686.67). Further adjustment by adding the correlated variables above into the multivariate model revealed that the SOFA score and prolonged delirium symptoms were independent predictors of mortality (HR = 1.32, *p* = 0.027). The mortality risk of the participants with prolonged delirium was 3.92 times higher than that of patients with no delirium (*p* = 0.049). The APACHE II score, ACCI, WASO, *r*24 and new‐onset delirium displayed no statistically significant correlations in the multivariate analysis. The overall Cox model was statistically significant (χ^2^ = 74.98, *p* < 0.001), showing that the model had good explanatory power with regard to participant survival.

**TABLE 6:** Cox regression analysis of factors influencing ICU patient survival (*N* = 74).

Independent variable	Univariate analysis		Multivariate analysis	
	HR (95% CI)	*p*	HR (95% CI)	*p*
Age (years)	0.99 (0.96–1.03)	0.766		
Ventilator (Yes vs. No)	31.19 (0.58–1686.67)	0.091		
Bedside window (Yes vs. No)	1.72 (0.64–4.62)	0.279		
MME (mg MME/day)	1.00 (1.00–1.004)	0.944		
Use of lorazepam (Yes vs. No)	0.64 (0.19–2.14)	0.468		
Use of midazolam (Yes vs. No)	1.64 (0.65–4.14)	0.293		
Use of dexmedetomidine (Yes vs. No)	0.21 (0.03–1.53)	0.122		
SOFA score	1.54 (1.35–1.76)	< 0.001	1.32 (1.03–1.68)	0.027
APACHE II score	1.15 (1.08–1.22)	< 0.001	0.98 (0.88–1.08)	0.626
MUST score	0.80 (0.49–1.30)	0.368		
ACCI	1.36 (1.14–1.62)	0.001	1.12 (0.82–1.53)	0.489
Katz index	0.95 (0.80–1.12)	0.542		
Length of hospital stay (days)	1.05 (1.00–1.11)	0.076		
Length of ICU stay (days)	1.00 (0.92–1.07)	0.909		
Albumin (g/dL)	0.74 (0.26–2.11)	0.571		
TST (min)	1.00 (0.99–1.01)	0.998		
WASO (min)	1.03 (1.02–1.05)	< 0.001	1.02 (1.00–1.03)	0.078
*r*24	0.01 (0.0001–0.02)	< 0.001	0.05 (0.001–2.63)	0.138
Changes in delirium state				
Prolonged delirium vs. no delirium	4.95 (1.52–16.09)	0.008	3.92 (1.01–15.26)	0.049
New‐onset delirium vs. no delirium	4.28 (1.36–13.46)	0.013	2.47 (0.64–9.51)	0.187

*Note:* Overall model summary: χ^2^ = 74.98, *p* < 0.001.

Abbreviations: 95% CI, 95% confidence interval; ACCI, age‐adjusted Charlson comorbidity index; APACHE II, acute physiology and chronic health evaluation II; HR, hazard ratio; MME, morphine milligram equivalents; MUST, malnutrition universal screening tool; *r*24, 24‐h autocorrelation coefficient; SOFA, sequential organ failure assessment; TST, total sleep time; WASO, wake after sleep onset.

## 4 Discussion

This study explored the correlations between changes in delirium state and overall prognosis in ICU patients using sleep and circadian rhythm monitoring indices. Our findings indicated high degrees of correlation among sleep disruption, rhythm dysregulation and delirium progression. Compared to those with no delirium, participants with prolonged delirium had not only higher SOFA scores, longer WASO and poorer *r*24 values but also significantly higher mortality risks. Those with new‐onset delirium also had higher SOFA scores and longer WASO than those with no delirium; however, their *r*24 values and mortality risks were not significantly different.

As an internationally recognised assessment tool for multiple organ failure, SOFA is widely applied in clinical practice and research and is an important foundation of mortality risk prediction for critically ill patients [47, 48]. In this study, SOFA scores were significantly higher in the prolonged delirium and new‐onset delirium groups than in the no delirium group, thereby indicating a possible correlation between illness severity and delirium. This is consistent with findings in existing literature that report organ failure as highly correlated with delirium risk [49]. Functional impairment in other organs (e.g., the lungs, liver or kidneys) can easily lead to metabolic imbalance or toxin accumulation in the brain, which causes acute confusion, that is, delirium [4, 50]. As the brain is a metabolically active organ and extremely sensitive to oxygen, glucose and metabolism, acute stress in ICU patients (e.g., septicaemia) could thus manifest as delirium [51].

Although many past studies have used TST to evaluate sleep conditions [52], this study found no significant correlation between TST and *r*24, which were our indices for circadian rhythm stability, suggesting that TST alone cannot sufficiently reflect sleep continuity and rhythm quality. In contrast, our discovery of a significant and negative correlation between WASO and *r*24 highly suggests that sleep fragmentation could disrupt physiological rhythms. Pillai et al. similarly reported that although TST is frequently used to assess sleep quality, sleep fragmentation is a more relevant index of circadian rhythm disruption [53]. Sleep disruption leads to poorer sleep quality, preventing adequate neural repair and cognitive integration [54]. In circumstances where patients had sufficient TST, sleep fragmentation still triggers immune responses in the central nervous system, releasing proinflammatory cytokines that perturb neurotransmission, affect synaptic plasticity and in turn damage cognitive functions [55]. Existing research has also indicated that acute sleep deprivation causes dysfunction in the hypothalamus‐brainstem reticular system, which subsequently affects the regulation of neurotransmitters (e.g., acetylcholine and dopamine) and leads to a decline in attention, memory and cognitive function, further exacerbating delirium symptoms [56].

After controlling the correlations between changes in delirium state and other factors in our bivariate polynomial logistic regression analysis, we found that a significant and negative correlation still existed between *r*24 and prolonged delirium, which thus indicates that circadian rhythm dysregulation is associated with persistent delirium. This result is consistent with that of a prospective cohort study of 76 ICU patients conducted by Li et al. [11] In that study, blood was drawn from patients three times daily to measure their melatonin and cortisol levels, and the Richard‐Campbell Sleep Questionnaire (RCSQ) and CAM‐ICU were used to assess their sleep and screen for delirium over 3 days. Their findings revealed that compared to those with no delirium, patients with delirium had lower melatonin and cortisol levels and lacked clear circadian rhythms, thereby supporting a strong correlation between the disruption of circadian rhythm and delirium. However, our bivariate polynomial logistic regression analysis found no significant correlation between *r*24 and new‐onset delirium, which may suggest that the patients in this group were still in the early stages of circadian rhythm disruption. In contrast, we found a correlation between WASO and new‐onset delirium; therefore, WASO could serve as an important basis for early delirium risk assessments and prevention interventions.

Common in ICU environments, noise and treatment activities (e.g., nighttime blood draws or repositioning) disturb the sleep quality and circadian rhythm of patients and perturb the functions of the central nervous system, such as impairing the brain's sense of direction, memory and attention, which in turn increases the risk of delirium and exacerbates disease deterioration [55]. The disruption of circadian rhythms reduces endogenous melatonin secretion and interferes with cortisol secretion rhythms. These thereby alter the ability of the immune system to maintain a stable neural environment in the brain and spinal cord, recognise and remove pathogens or damage signals and regulate the dynamic balance between proinflammatory and anti‐inflammatory responses [57]. Circadian rhythm dysregulation therefore not only reduces the adaptability of the brain to external stimuli and increases the risk of neuroinflammation, but it is also a crucial mechanism of delirium exacerbation. The Cox regression analysis in the present study revealed that WASO and *r*24 each had high predictive power for the mortality risk of participants in the univariate analysis. Past studies have further indicated that good sleep quality and stable circadian rhythms help to maintain immune functions, metabolic balance and cardiovascular and neuroendocrine regulation, thereby reducing inflammation and mortality rates in critically ill patients [58, 59]. However, neither WASO nor *r*24 was significant in our multivariate Cox regression analysis, due perhaps to the limited sample size and interaction effects.

Common delirium risk factors such as sedatives, analgesics and environmental factors were not significant in the analyses in this study, which could be attributed to the limited sample size. Current guidelines suggest prioritising dexmedetomidine when using sedatives. However, as for whether benzodiazepines should be used to treat patient anxiety, no experts have put forward any concrete suggestions [60]. Midazolam is a short‐acting benzodiazepine used to enhance inhibitory neurotransmission and achieve sedative, anxiolytic and amnestic effects. Excessive use of this drug can inhibit central nervous system activity, causing confusion and disorientation [61]. Midazolam also disrupts normal sleep structure and inhibits deep sleep and rapid eye movement sleep, which leads to sleep fragmentation and circadian rhythm dysregulation and increases the risk of delirium [62]. With regard to drug use, this study found no significant differences in the proportions of lorazepam, midazolam and dexmedetomidine used in the three groups; however, a relatively high percentage (25.0%) of the participants in the prolonged delirium group were taking midazolam, which may indicate a potential correlation between midazolam use and prolonged delirium. Though due to sample size limitations, further research is required to elucidate the effects of midazolam.

This study revealed that mortality risks were significantly higher in the prolonged delirium and new‐onset delirium groups than in the no delirium group. Although the participants in the new‐onset delirium group displayed no delirium on the first 2 days, their mortality risk was very similar to that of the prolonged delirium (45.0% vs. 45.8%); thus, this finding stresses the importance of dynamically tracking changes in states of delirium. Even if delirium is acute, it may reflect exacerbation in the overall condition or a potentially critical situation [14]. Acute delirium often manifests as an early sign of deteriorating physical conditions, such as a neurocognitive response triggered by infection, dehydration, hypoxia, medication side effects or other acute stressors. Thus, delirium is not only a manifestation of confusion but also a possible warning that signals systematic deterioration and requires early detection and intervention measures [1]. Furthermore, this study found that the risk of mortality of participants in the prolonged delirium group was over three times higher than that of participants in the no delirium group after controlling for the SOFA score, APACHE II score, ACCI, WASO and *r*24. Ely et al. analysed the delirium in 275 ICU patients using CAM‐ICU; after controlling for age and illness severity, a multivariate analysis indicated delirium as an independent predictor of mortality [63]. The mortality rate of the delirium patients in their study was also significantly higher. Tsui et al. further noted that prolonged delirium and long‐term cognitive impairment are associated with functional decline and mortality rate [64].

## 5 Limitations

Note that this study involved a single medical centre, which may limit the external validity of the results. Large‐scale, multi‐centre studies should be conducted in the future to further investigate the possible effectiveness of measures aimed at improving sleep quality and circadian rhythm on delirium. Light exposure is known to influence circadian rhythm and sleep. In this study, the presence of a window was used as a proxy for environmental light exposure due to the challenges of continuous monitoring in the ICU. While consistent with prior studies, this approach may have introduced variability in actual exposure levels and should be acknowledged as a limitation. Future research could incorporate objective light sensors to provide precise quantification.

## 6 Implications and Recommendations for Practice

We suggest that circadian rhythms and sleep quality be incorporated into routine monitoring and risk assessments in ICU patient care and that suitable non‐pharmacological intervention strategies (e.g., optimising lighting, minimising nighttime disturbances and regularising daily routines) be developed to prevent delirium exacerbation and increase survival.

## 7 Conclusions

This study demonstrated that the sleep quality and circadian rhythm stability of ICU patients is closely associated with changes in delirium state. Sleep disruption and circadian rhythm dysregulation may further affect the prognoses of patients by inducing delirium. From our findings, we can infer that reductions in sleep fragmentation and the maintenance of circadian rhythm stability could assist in lowering delirium incidence and improving clinical prognoses.

## Ethics Statement

This study was approved by the Taipei Medical University–Joint Institutional Review Board (TMU‐Joint IRB) (Review No.: N202404040) on April 30, 2024, and was conducted in accordance with the Declaration of Helsinki and related ethical guidelines.

## Consent

This study was an observational study with no invasive measures. The details of this study were explained by the researchers or the primary nurses to the participants when their conditions were stable, and patients were only included in this study after written consent was obtained from the patients themselves or their legal representatives. In the consent forms, the researchers clearly expressed that participation was voluntary and that the participants could choose to withdraw at any time without affecting their healthcare rights. All collected data were anonymised to protect the privacy of the participants. The data were stored in a password‐protected computer, and only research team members could access and analyse the data as authorised. All physiological data, such as the sleep parameters, were managed in the same way to prevent data leaks. This study did not involve any financial incentives or risky interventions, and all procedures prioritised the respect and protection of patient rights.

## Conflicts of Interest

The authors declare no conflicts of interest.

## Data Availability

The data that support the findings of this study are available on request from the corresponding author. The data are not publicly available due to privacy or ethical restrictions.

## References

[1] J. E. Wilson , M. F. Mart , C. Cunningham , et al., “Delirium,” Nature Reviews Disease Primers 6, no. 1 (2020): 90, 10.1038/s41572-020-00223-4. PMC9012267 33184265

[2] K. Callahan , S. Chesnut , and S. Lasiter , “Prevalence of Clinical Factors Experienced by Patients Who Developed Delirium in Intensive Care Units: A Descriptive Study Involving 20,000 Patients,” Delirium Communications (2024), 10.56392/001c.124514.

[3] G. U. Johnson , T. B. Amanda , M. Christopher , and E. Beverley , “Delirium Prevention and Management in an Adult Intensive Care Unit Through Evidence–Based Nonpharmacological Interventions: A Scoping Review,” Collegian 31, no. 4 (2024): 232–251, 10.1016/j.colegn.2024.05.001.

[4] J. L. Stollings , K. Kotfis , G. Chanques , B. T. Pun , P. P. Pandharipande , and E. W. Ely , “Delirium in Critical Illness: Clinical Manifestations, Outcomes, and Management,” Intensive Care Medicine 47, no. 10 (2021): 1089–1103, 10.1007/s00134-021-06503-1. 34401939 PMC8366492

[5] K. van der Wulp , M. H. van Wely , M. J. P. Rooijakkers , et al., “Delirium After TAVR: Crosspassing the Limit of Resilience,” JACC Cardiovascular Interventions 13, no. 21 (2020): 2453–2466, 10.1016/j.jcin.2020.07.044. 33153562

[6] A. Taha , H. Xu , R. Ahmed , et al., “Medical and Economic Burden of Delirium on Hospitalization Outcomes of Acute Respiratory Failure: A Retrospective National Cohort,” Medicine (Baltimore) 102, no. 2 (2023): e32652, 10.1097/MD.0000000000032652. 36637939 PMC9839276

[7] M. P. Knauert , N. T. Ayas , K. J. Bosma , et al., “Causes, Consequences, and Treatments of Sleep and Circadian Disruption in the ICU: An Official American Thoracic Society Research Statement,” American Journal of Respiratory and Critical Care Medicine 207, no. 7 (2023): e49–e68, 10.1164/rccm.202301-0184ST. 36999950 PMC10111990

[8] I. Telias and M. E. Wilcox , “Sleep and Circadian Rhythm in Critical Illness,” Critical Care 23, no. 1 (2019): 82, 10.1186/s13054-019-2366-0. 30850003 PMC6408803

[9] J. Kang and J. Kang , “Sleep Characteristics of Patients Admitted to Intensive Care Units After Major Abdominal Surgery,” Journal of Korean Critical Care Nursing 17, no. 3 (2024): 50–61.

[10] K. E. Schwab , B. Ronish , D. M. Needham , A. Q. To , J. L. Martin , and B. B. Kamdar , “Actigraphy to Evaluate Sleep in the Intensive Care Unit. A Systematic Review,” Annals of the American Thoracic Society 15, no. 9 (2018): 1075–1082, 10.1513/AnnalsATS.201801-004OC. 29944386 PMC6322043

[11] J. Li , S. Cai , X. Liu , et al., “Circadian Rhythm Disturbance and Delirium in ICU Patients: A Prospective Cohort Study,” BMC Anesthesiology 23, no. 1 (2023): 203, 10.1186/s12871-023-02163-4. 37312021 PMC10262569

[12] M. Y. Chong , K. G. Frenken , S. J. P. M. Eussen , et al., “Longitudinal Associations of Diurnal Rest–Activity Rhythms With Fatigue, Insomnia, and Health–Related Quality of Life in Survivors of Colorectal Cancer up to 5 Years Post–Treatment,” International Journal of Behavioral Nutrition and Physical Activity 21, no. 1 (2024): 51, 10.1186/s12966-024-01601-x. 38698447 PMC11067118

[13] P. S. Lee , Y. L. Liu , Y. L. Chen , and W. J. Cheng , “Work Is Associated With a More Robust Rest–Activity Rhythm and High‐Intensity Physical Activity Among Older Adults,” Aging Clinical and Experimental Research 37, no. 1 (2025): 200, 10.1007/s40520-025-03083-8. 40580344 PMC12206208

[14] M. Tachibana and T. Inada , “Poor Prognostic Impact of Delirium: Especially on Mortality and Institutionalisation,” Psychogeriatrics 23, no. 1 (2023): 187–195, 10.1111/psyg.12914. 36416212

[15] Y. Aikawa , S. Ogata , S. Honda , et al., “Prolonged Delirium During Hospitalization Is Associated With Worse Long–Term and Short–Term Outcomes in Patients With Acute Heart Failure,” International Journal of Cardiology 399 (2024): 131776, 10.1016/j.ijcard.2024.131776. 38216062

[16] Y. Huang , Y. Gao , S. Quan , et al., “Development and Internal–External Validation of the ACCI–SOFA Model for Predicting in–Hospital Mortality of Patients With Sepsis‐3 in the ICU: A Multicenter Retrospective Cohort Study,” Shock 61, no. 3 (2024): 367–374, 10.1097/SHK.0000000000002311. 38407987

[17] Y. Tian , Y. Yao , J. Zhou , et al., “Dynamic APACHE II Score to Predict the Outcome of Intensive Care Unit Patients,” Frontiers in Medicine (Lausanne) 8 (2022): 744907, 10.3389/fmed.2021.744907. PMC8826444 35155461

[18] E. E. Jonescu , E. Litton , and B. Farrell , “Investigating the Interplay of Thermal, Lighting, and Acoustics in Intensive Care for Enhanced Patient Well–Being and Clinical Outcomes,” HERD 18, no. 2 (2025): 362–377, 10.1177/19375867251317235. 39957004 PMC12050381

[19] J. L. Vincent , M. J. Dubois , R. J. Navickis , and M. M. Wilkes , “Hypoalbuminemia in Acute Illness: Is There a Rationale for Intervention? A Critical Review of the Literature,” Annals of Surgery 237, no. 3 (2003): 319–334, 10.1097/01.SLA.0000055547.93484.87. 12616115 PMC1514323

[20] A. R. Al Huraizi , J. S. Al‐Maqbali , R. S. Al Farsi , et al., “Delirium and Its Association With Short– And Long–Term Health Outcomes in Medically Admitted Patients: A Prospective Study,” Journal of Clinical Medicine 12, no. 16 (2023): 5346, 10.3390/jcm12165346. 37629388 PMC10455146

[21] M. C. B. Adams , K. A. Sward , M. L. Perkins , and R. W. Hurley , “Standardizing Research Methods for Opioid Dose Comparison: The NIH HEAL Morphine Milligram Equivalent Calculator,” Pain 166, no. 8 (2025): 1729–1737, 10.1097/j.pain.0000000000003529. 39907478 PMC12266977

[22] E. W. Ely , B. Truman , A. Shintani , et al., “Monitoring Sedation Status Over Time in ICU Patients: Reliability and Validity of the Richmond Agitation‐Sedation Scale (RASS),” Journal of the American Medical Association 289, no. 22 (2023): 2983–2991, 10.1001/jama.289.22.2983. 12799407

[23] C. N. Sessler , M. S. Gosnell , M. J. Grap , et al., “The Richmond Agitation‐Sedation Scale: Validity and Reliability in Adult Intensive Care Unit Patients,” American Journal of Respiratory and Critical Care Medicine 166, no. 10 (2002): 1338–1344, 10.1164/rccm.2107138. 12421743

[24] A. Li , L. Ling , H. Qin , et al., “Prognostic Evaluation of Quick Sequential Organ Failure Assessment Score in ICU Patients With Sepsis Across Different Income Settings,” Critical Care 28, no. 1 (2024): 30, 10.1186/s13054-024-04804-7. 38263076 PMC10804657

[25] R. Moreno , A. Rhodes , L. Piquilloud , et al., “The Sequential Organ Failure Assessment (SOFA) Score: Has the Time Come for an Update?,” Critical Care 27, no. 1 (2023): 15, 10.1186/s13054-022-04290-9. 36639780 PMC9837980

[26] F. L. Ferreira , D. P. Bota , A. Bross , C. Mélot , and J. L. Vincent , “Serial Evaluation of the SOFA Score to Predict Outcome in Critically Ill Patients,” Journal of the American Medical Association 286, no. 14 (2001): 1754–1758, 10.1001/jama.286.14.1754. 11594901

[27] S. Lemeshow , D. Teres , J. Klar , J. S. Avrunin , S. H. Gehlbach , and J. Rapoport , “Mortality Probability Models (MPM II) Based on an International Cohort of Intensive Care Unit Patients,” Journal of the American Medical Association 270, no. 20 (1993): 2478–2486. 8230626

[28] N. Tewari , J. Rodrigues , L. Bothamley , N. Altaf , and S. Awad , “The Utilisation of the MUST Nutritional Screening Tool on Vascular Surgical Wards,” BMJ Quality Improvement Reports 2, no. 1 (2013): u201374.w1122, 10.1136/bmjquality.u201374.w1122. PMC4652729 26734198

[29] Y. Sharma , C. Thompson , B. Kaambwa , R. Shahi , and M. Miller , “Validity of the Malnutrition Universal Screening Tool (MUST) in Australian Hospitalized Acutely Unwell Elderly Patients,” Asia Pacific Journal of Clinical Nutrition 26, no. 6 (2017): 994–1000, 10.6133/apjcn.022017.15. 28917223

[30] M. E. Charlson , P. Pompei , K. L. Ales , and C. R. MacKenzie , “A New Method of Classifying Prognostic Comorbidity in Longitudinal Studies: Development and Validation,” Journal of Chronic Diseases 40, no. 5 (1987): 373–383, 10.1016/0021-9681(87)90171-8. 3558716

[31] S. Katz , T. D. Downs , H. R. Cash , and R. C. Grotz , “Progress in Development of the Index of ADL,” Gerontologist 10 (1970): 20–30, 10.1093/geront/10.1_part_1.20. 5420677

[32] N. Rathnayake , R. Karunadasa , T. Abeygunasekara , W. De Zoysa , D. Palangasinghe , and S. Lekamwasam , “Katz Index of Activities of Daily Living in Assessing Functional Status of Older People: Reliability and Validity of Sinhala Version,” Dialogues Health 2 (2023): 100134, 10.1016/j.dialog.2023.100134. 38515463 PMC10953911

[33] E. W. Ely , S. K. Inouye , G. R. Bernard , et al., “Delirium in Mechanically Ventilated Patients: Validity and Reliability of the Confusion Assessment Method for the Intensive Care Unit (CAM‐ICU),” Journal of the American Medical Association 286, no. 21 (2001): 2703–2710, 10.1001/jama.286.21.2703. 11730446

[34] F. Miranda , F. Gonzalez , M. N. Plana , J. Zamora , T. J. Quinn , and P. Seron , “Confusion Assessment Method for the Intensive Care Unit (CAM‐ICU) for the Diagnosis of Delirium in Adults in Critical Care Settings,” Cochrane Database of Systematic Reviews 11, no. 11 (2023): CD013126, 10.1002/14651858.CD013126.pub2. 37987526 PMC10661047

[35] B. A. Khan , A. J. Perkins , S. Gao , et al., “The Confusion Assessment Method for the ICU–7 Delirium Severity Scale: A Novel Delirium Severity Instrument for Use in the ICU,” Critical Care Medicine 45, no. 5 (2017): 851–857, 10.1097/CCM.0000000000002368. 28263192 PMC5392153

[36] S. Liu , J. J. Schlesinger , A. B. McCoy , et al., “New Onset Delirium Prediction Using Machine Learning and Long Short–Term Memory (LSTM) in Electronic Health Record,” Journal of the American Medical Informatics Association 30, no. 1 (2022): 120–131, 10.1093/jamia/ocac210. 36303456 PMC9748586

[37] C. J. Slor , J. Witlox , D. Adamis , et al., “Predicting Delirium Duration in Elderly Hip–Surgery Patients: Does Early Symptom Profile Matter?,” Current Gerontology and Geriatrics Research 2013 (2013): 962321, 10.1155/2013/962321. 23533395 PMC3600209

[38] D. Fekedulegn , M. E. Andrew , M. Shi , J. M. Violanti , S. Knox , and K. E. Innes , “Actigraphy‐Based Assessment of Sleep Parameters,” Annals of Work Exposures and Health 64, no. 4 (2020): 350–367, 10.1093/annweh/wxaa007. 32053169 PMC7191872

[39] P. Gupta , J. L. Martin , D. M. Needham , S. Vangala , E. Colantuoni , and B. B. Kamdar , “Use of Actigraphy to Characterize Inactivity and Activity in Patients in a Medical ICU,” Heart & Lung 49, no. 4 (2020): 398–406, 10.1016/j.hrtlng.2020.02.002. 32107065 PMC7305977

[40] K. E. Schwab , A. Q. To , J. Chang , et al., “Actigraphy to Measure Physical Activity in the Intensive Care Unit: A Systematic Review,” Journal of Intensive Care Medicine 35, no. 11 (2020): 1323–1331, 10.1177/0885066619863654. 31331220 PMC7449762

[41] C. Labrosciano , R. Tavella , A. Reynolds , et al., “The Association Between Sleep Duration and Quality With Readmissions: An Exploratory Pilot‐Study Among Cardiology Inpatients,” Clocks Sleep 2, no. 2 (2020): 120–142, 10.3390/clockssleep2020011. 33089196 PMC7445848

[42] B. B. Kamdar , D. M. Needham , and N. A. Collop , “Sleep Deprivation in Critical Illness: Its Role in Physical and Psychological Recovery,” Journal of Intensive Care Medicine 27, no. 2 (2012): 97–111, 10.1177/0885066610394322. 21220271 PMC3299928

[43] E. Gottlieb , L. Churilov , E. Werden , et al., “Sleep‐Wake Parameters Can be Detected in Patients With Chronic Stroke Using a Multisensor Accelerometer: A Validation Study,” Journal of Clinical Sleep Medicine 17, no. 2 (2021): 167–175, 10.5664/jcsm.8812. 32975195 PMC7853221

[44] Y. Asaka and C. Kondo , “A Longitudinal Study on Parent‐Infant Rest‐Activity Rhythms Using Actigraphy From Late Pregnancy to Eight Months After Birth,” Cureus 16, no. 12 (2024): e76029, 10.7759/cureus.76029. 39835042 PMC11743615

[45] S. J. Jaiswal , S. R. S. Bagsic , E. Takata , B. B. Kamdar , S. Ancoli‐Israel , and R. L. Owens , “Actigraphy‐Based Sleep and Activity Measurements in Intensive Care Unit Patients Randomized to Ramelteon or Placebo for Delirium Prevention,” Scientific Reports 13, no. 1 (2023): 1450, 10.1038/s41598-023-28095-0. 36702822 PMC9879948

[46] J. M. Hendricks , J. R. Metz , H. M. Boss , R. W. J. Collin , E. de Vrieze , and E. van Wijk , “Actigraphy–Based Assessment of Circadian Rhythmicity and Sleep in Patients With Usher Syndrome Type 2a: A Case‐Control Study,” Journal of Sleep Research 34, no. 4 (2025): e14456, 10.1111/jsr.14456. 39740053 PMC12215243

[47] E. Mousabeygi , M. Rahmati , N. Salari , and R. Jalali , “Predicting Mortality of Elderly Patients Undergoing Open Heart Surgery With Delirium Using Sequential Organ Failure Assessment (SOFA), multi–Organ Dysfunction (MODS), and Logistic Organ Dysfunction System (LODS) Scores,” Geriatric Nursing 60 (2024): 146–149, 10.1016/j.gerinurse.2024.08.023. 39244800

[48] S. Sekhar , V. Pratap , K. Gaurav , et al., “The Value of the Sequential Organ Failure Assessment (SOFA) Score and Serum Lactate Level in Sepsis and Its Use in Predicting Mortality,” Cureus 15, no. 7 (2023): e42683, 10.7759/cureus.42683. 37649942 PMC10464653

[49] J. Yang , Y. Zhou , Y. Kang , et al., “Risk Factors of Delirium in Sequential Sedation Patients in Intensive Care Units,” BioMed Research International 2017 (2017): 3539872, 10.1155/2017/3539872. 29226131 PMC5684530

[50] E. F. M. Wijdicks , “Uremia and the Brain: The Contentious History of a Small Molecule,” Neurocritical Care (2024), 10.1007/s12028-024-02157-1. 39505791

[51] J. Li , Q. Jia , L. Yang , et al., “Sepsis‐Associated Encephalopathy: Mechanisms, Diagnosis, and Treatments Update,” International Journal of Biological Sciences 21, no. 7 (2025): 3214–3228, 10.7150/ijbs.102234. 40384873 PMC12080397

[52] J. Zitser , I. E. Allen , N. Falgàs , et al., “Pittsburgh Sleep Quality Index (PSQI) Responses Are Modulated by Total Sleep Time and Wake After Sleep Onset in Healthy Older Adults,” PLoS One 17, no. 6 (2022): e0270095, 10.1371/journal.pone.0270095. 35749529 PMC9232154

[53] J. A. Pillai , J. Bena , L. M. Bekris , et al., “Unique Sleep and Circadian Rhythm Dysfunction Neuroinflammatory and Immune Profiles in Alzheimer's Disease With Mild Cognitive Impairment,” Journal of Alzheimer's Disease 81, no. 2 (2021): 487–492, 10.3233/JAD-201573. PMC8179975 33814445

[54] S. J. Jaiswal , D. Y. Kang , N. E. Wineinger , and R. L. Owens , “Objectively Measured Sleep Fragmentation Is Associated With Incident Delirium in Older Hospitalized Patients: Analysis of Data Collected From an Randomized Controlled Trial,” Journal of Sleep Research 30, no. 3 (2021): e13205, 10.1111/jsr.13205. 33051948

[55] L. Showler , Y. Ali Abdelhamid , J. Goldin , and A. M. Deane , “Sleep During and Following Critical Illness: A Narrative Review,” World Journal of Critical Care Medicine 12, no. 3 (2023): 92–115, 10.5492/wjccm.v12.i3.92. 37397589 PMC10308338

[56] Y. Y. Fan , R. Y. Luo , M. T. Wang , C. Y. Yuan , Y. Y. Sun , and J. Y. Jing , “Mechanisms Underlying Delirium in Patients With Critical Illness,” Frontiers in Aging Neuroscience 16 (2024): 1446523, 10.3389/fnagi.2024.1446523. 39391586 PMC11464339

[57] R. Chen , B. N. Routh , A. D. Gaudet , and L. K. Fonken , “Circadian Regulation of the Neuroimmune Environment Across the Lifespan: From Brain Development to Aging,” Journal of Biological Rhythms 38, no. 5 (2023): 419–446, 10.1177/07487304231178950. 37357738 PMC10475217

[58] M. Henríquez‐Beltrán , I. D. Benítez , R. Vaca , et al., “The Trajectory of Sleep After Critical Illness: A 24–Month Follow‐Up Study,” Annals of Intensive Care 15, no. 1 (2025): 28, 10.1186/s13613-025-01449-9. 40019644 PMC11871202

[59] K. Zheng , M. Wu , Y. Cao , et al., “Circadian Syndrome and Mortality Risk in Adults Aged ≥ 40 Years: A Prospective Cohort Analysis of CHARLS and NHANES,” Scientific Reports 15, no. 1 (2025): 14791, 10.1038/s41598-025-99631-3. 40295832 PMC12037739

[60] K. Lewis , M. C. Balas , J. L. Stollings , et al., “A Focused Update to the Clinical Practice Guidelines for the Prevention and Management of Pain, Anxiety, Agitation/ Sedation, Delirium, Immobility, and Sleep Disruption in Adult Patients in the ICU,” Critical Care Medicine 53, no. 3 (2025): e711–e727, 10.1097/CCM.0000000000006574. 39982143

[61] J. U. Peter , P. Dieudonné , and O. Zolk , “Pharmacokinetics, Pharmacodynamics, and Side Effects of Midazolam: A Review and Case Example,” Pharmaceuticals (Basel) 17, no. 4 (2024): 473, 10.3390/ph17040473. 38675433 PMC11054797

[62] H. J. Shi , R. X. Yuan , J. Z. Zhang , J. H. Chen , and A. M. Hu , “Effect of Midazolam on Delirium in Critically Ill Patients: A Propensity Score Analysis,” Journal of International Medical Research 50, no. 4 (2022): 3000605221088695, 10.1177/03000605221088695. 35466751 PMC9044793

[63] E. W. Ely , A. Shintani , B. Truman , et al., “Delirium as a Predictor of Mortality in Mechanically Ventilated Patients in the Intensive Care Unit,” JAMA 291, no. 14 (2004): 1753–1762, 10.1001/jama.291.14.1753. 15082703

[64] A. Tsui , S. D. Searle , H. Bowden , et al., “The Effect of Baseline Cognition and Delirium on Long–Term Cognitive Impairment and Mortality: A Prospective Population–Based Study,” Lancet Healthy Longevity 3, no. 4 (2022): e232–e241, 10.1016/S2666-7568(22)00013-7. 35382093 PMC7612581

